# The Behavior of Polymeric Pipes in Drinking Water Distribution System—Comparison with Other Pipe Materials

**DOI:** 10.3390/polym15193872

**Published:** 2023-09-24

**Authors:** Daniela Simina Stefan, Magdalena Bosomoiu, Georgeta Teodorescu

**Affiliations:** 1 Department of Analytical Chemistry and Environmental Engineering, Faculty of Chemical Engineering and Biotechnologies, National University of Science and Technology Politehnica of Bucharest, 1-7 Polizu Street, 011061 Bucharest, Romania; daniela.stefan@upb.ro (D.S.S.); diaconescu_gina@yahoo.com (G.T.); 2 Doctoral School, Specialization of Environmental Engineering, Faculty of Chemical Engineering and Biotechnologies, National University of Science and Technology Politehnica of Bucharest, 1-7 Polizu Street, 011061 Bucharest, Romania

**Keywords:** tap water, polymeric pipe, concrete pipe, iron pipe, lead pipe, leaching, corrosion

## Abstract

The inner walls of the drinking water distribution system (DWDS) are expected to be clean to ensure a safe quality of drinking water. Complex physical, chemical, and biological processes take place when water comes into contact with the pipe surface. This paper describes the impact of leaching different compounds from the water supply pipes into drinking water and subsequent risks. Among these compounds, there are heavy metals. It is necessary to prevent these metals from getting into the DWDS. Those compounds are susceptible to impacting the quality of the water delivered to the population either by leaching dangerous chemicals into water or by enhancing the development of microorganism growth on the pipe surface. The corrosion process of different pipe materials, scale formation mechanisms, and the impact of bacteria formed in corrosion layers are discussed. Water treatment processes and the pipe materials also affect the water composition. Pipe materials act differently in the flowing and stagnation conditions. Moreover, they age differently (e.g., metal-based pipes are subjected to corrosion while polymer-based pipes have a decreased mechanical resistance) and are susceptible to enhanced bacterial film formation. Water distribution pipes are a dynamic environment, therefore, the models that are used must consider the changes that occur over time. Mathematical modeling of the leaching process is complex and includes the description of corrosion development over time, correlated with a model for the biofilm formation and the disinfectants–corrosion products and disinfectants–biofilm interactions. The models used for these processes range from simple longitudinal dispersion models to Monte Carlo simulations and 3D modeling. This review helps to clarify what are the possible sources of compounds responsible for drinking water quality degradation. Additionally, it gives guidance on the measures that are needed to maintain stable and safe drinking water quality.

## 1. Introduction

Water is essential for life development throughout the world, all plants and animals need water. Its quality (expressed by chemical, physical and biological characteristics) influences the population’s health. Drinking water quality varies from place to place and is affected by the water source, the treatment that water undergoes, and the pipe distribution system.

Water in contact with the pipe material can cause component leaching, pipe corrosion over time, or promote microorganism growth on the inner pipe surface. The first two mechanisms lead to chemical contamination of water whilst microorganism multiplication deteriorates the biological stability of water (development of pathogens, deterioration of taste, odor, and color). Water quality at the tap and the problems that arise are specific to each water distribution system. The 2021 EU Directive regarding the quality of drinking water is stricter regarding the presence of toxic chemical compounds [1].

Microorganism growth inside the pipes depends on several factors, such as availability of nutrients, concentration of disinfectant, water temperature and pH, existence of biofilm or corrosion layer on the pipes’ inner surface.

The biological stability of drinking water is defined as the capacity of water to maintain the same microbiological characteristics from the treatment plant to the consumption point. The presence of bacterial communities during the water treatment step is carefully checked. However, the distribution system can contribute to the development of microorganisms in the biofilm and corrosion layers formed on the pipes’ inner walls [2]. The adhesion of microorganisms is influenced by several factors: (i) pipe characteristics (such as material, roughness), (ii) water physical-chemical parameters (pH, temperature, load in organic compounds, existence of minerals), and (iii) characteristics of microorganisms (ability to produce extracellular polymeric substance, cell hydrophobicity and motility) [3,4,5,6,7].

Among the first materials used for the water distribution systems (concrete and lead), lead was extensively employed. Lead exposure has severe consequences (affecting mental and physical development), with symptoms that do not appear until dangerous amounts have accumulated in the human body [8]. Adverse health effects have also been observed in the prenatal stage [9]. The lead limit in drinking water has been decreased over time to 10 μg/L in the European Union in compliance with the value recommended by the WHO, but the aim is to reduce the limit to 5 μg/L in the next 15 years [10,11]. Because there is no natural source of lead in drinking water, the only way to achieve the new limit is by replacing the lead pipes. The US EPA proposed the complete elimination of lead (concentration of 0 μg/L) from drinking water [12]. Because of that, during the last decades, it was decided to replace lead plumbing with other materials such as iron, galvanized steel, PVC, and copper [13,14,15,16,17]. Each of these materials has advantages and disadvantages that will be further discussed.

Aluminum can be leached from cement-based pipes. Constant exposure to this ion can lead to an important risk of cognitive decline, dementia, and Alzheimer’s disease [18,19]. In New York, no correlation was found between the asbestos contained in drinking water and cancer occurrence among the population [20]. However, asbestos fibers in drinking water could become airborne and enter the lungs. Another study carried out in Norway supports the hypothesis of an association between ingested asbestos via drinking water and gastrointestinal cancer risk [21]. By now, there are few studies that link the ingestion of asbestos fibers with gastrointestinal cancer. For this reason, the European Union did not establish a concentration limit for asbestos in drinking water. US EPA has set a limit of seven million asbestos fibers per liter of water [22]. However, a recent study by ANSES (French Agency for Food, Environmental, and Occupational Health and Safety) stated that the link between gastrointestinal cancer occurrence and ingestion of asbestos fibers is underestimated [23].

Galvanized pipes are a source of zinc in drinking water. Iron pipes are a source of arsenic. Zinc, arsenic, and copper are heavy metals that pose health risk issues, and their concentrations must be monitored in DWDS [24,25,26,27,28,29]. WHO has stated that although some epidemiological studies lack precision in the evaluation of exposure and the methodology development, there is clear evidence that cancer development is related to the consumption of elevated levels of drinking water contaminated with arsenic [30]. Chromium has negative effects at the neurological and reproductive levels, but its toxicity depends on its valence: Hexavalent chromium is more toxic than trivalent chromium [31,32,33]. Cadmium is susceptible to accumulation in kidneys, its limit, as proposed by WHO, is 3 μg/L [34].

The health problems depending on the nature of the contaminant are summarized in Table 1.

When a pipe corrodes, it leaches substances that accumulate in drinking water, but also, the pipe softens and loses its mechanical strength, overall reducing its service life.

Modern manufacturing technologies allow the fabrication of water distribution pipes by combining different materials (e.g., ductile iron pipe with cement lining or polymer lining, such as polyethylene or polyurethane). However, the behavior of these pipes regarding the progressive release of dangerous compounds due to pipe corrosion and aging is not entirely known.

## 2. Materials Used to Manufacture Drinking Water Distribution Pipes 

### 2.1. Lead

The main lead source in a lead pipe is the pipe itself. Besides lead-manufactured pipes, another source of lead in non-lead pipe systems are brass fixtures and lead-solder connections [41,42,43,44,45]. These fittings represent discrete sources of lead that may give rise to significant lead concentrations in drinking water. There is no safe lead concentration in drinking water. Lead concentration can vary within a home, at the same tap over time, or between homes. Currently, new lead systems are no longer installed but many older DWDS still contain lead pipes. In recent years, lead pipes have been gradually replaced worldwide, especially in the cases where Pb concentrations are high. 

Concentrations higher than 10 μg/L were consistently measured in old pipe systems in Taiwan, which represents a health risk for the population [46].

However, care must be taken when replacing parts of the lead pipe system. In Canada, replacement of about 80% of lead pipe with copper pipe caused sustained lead release (sometimes worse than a full lead system) up to 12 weeks upon substitution [47]. The same behavior has been reported in the USA [48]. This was attributed to galvanic corrosion between aged lead pipes and new copper pipes [47,49].

The factors that control the lead release from corrosion scale are [47,50,51,52,53]:

(a) Water chemistry parameters:
  (i) pH, alkalinity
  (ii) The content of dissolved inorganic carbon
  (iii) Presence of disinfectants: chlorine, chloramine, dissolved oxygen
  (iv) Presence of corrosion inhibitors (orthophosphate, orthophosphate/polyphosphate mixture, silica)
  (v) Oxidation Reduction Potential
  (vi) Presence of organic matter
(b) Flowing regime: alternate flow/stagnant regime; pipes flushing.

Lead scale is a complex structure that accumulates many different metallic compounds, often in different layers. Because of that, lead release into the flowing water is more complex than the corresponding solubility mechanism. One of the factors affecting the lead leaching from the corrosion scale is the pH. Lead solubility from the scale increases with decreasing pH, allowing the detachment of lead particles from the scale into drinking water [54,55]. Experiments have shown that arsenic and aluminum concentrations in drinking water during the pH experiments are in strong correlation. This suggests that the aluminosilicate present in the scale accumulates arsenic that can be later released when conditions are favorable [50,56].

**Figure 1:**
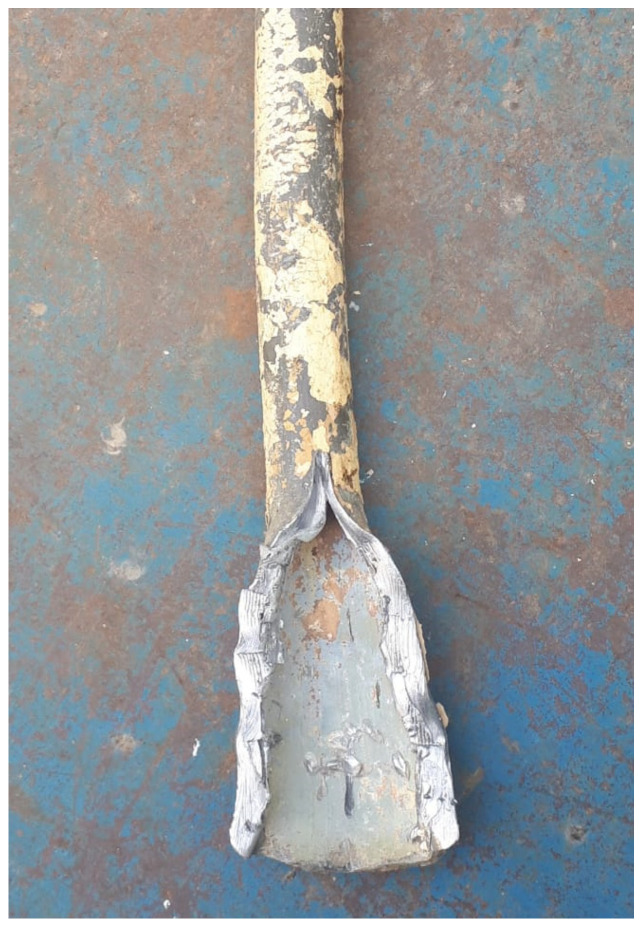
Lead pipe replaced after about 50 years in service (courtesy of Ecoaqua S.A. Călăraşi, Romania).

The lead corrosion scale can contain other heavy metals, such as V, Sb, Cu, Mn, and Cr [56,57,58,59,60]. In total, 2.8% of water samples analyzed in a DWDS in USA exceeded the maximum allowable concentration for Pb, As, and Cu [60]. Characterization methods showed that hydrocerussite phase (Pb_3_(CO_3_)_2_(OH)_2_) is the major lead crystalline corrosion compound in the scale [51,56]. Cerussite (PbCO_3_) was also present in some cases. In the inner scale layers, PbO_2_ and Pb_3_O_4_ were present [56]. Lead dioxide acts as a protective layer. The formation of PbO_2_ was reported to be beneficial in terms of pipe protection against future corrosion [61]. Metallic lead reacts with water only in the presence of oxidizing compounds to form lead hydroxide, Pb(OH)_2_ [62]. Then, white lead, an alkalic lead carbonate compound with high toxicity (hydrocerussite, 2PbCO_3_·Pb(OH)_2_) forms (Figure 1). The addition of orthophosphate decreases the dissolution rate of hydrocerussite [51]. Darker layers correspond to accumulation in the lead scale of manganese-containing compounds [58].

In soft water, traces of lead form colloidal lead compounds that are difficult to remove [63]. It has been experimentally observed that higher lead levels in water are often accompanied by high iron concentrations [64]. Lead with iron and natural organic matter present in the environment forms colloidal particles that are easily detached and transported in the DWDS. This was confirmed by size exclusion chromatography with UV and multi-element (ICP-MS) detection. Lead is mobilized via adsorption to iron colloidal particles.

The change of disinfectant from chlorine to chloramine caused an increase in lead corrosion. This is because the predominantly tetravalent lead (PbO_2_) scale is destabilized in the presence of chloramine [65].

Analysis of tubercle scales collected from pipelines in Columbus, Ohio, found they contained microorganisms, such as sulfate reducers, nitrate reducers, nitrite oxidizers, ammonia oxidizers, and sulfur oxidizers [66].

To help reduce lead concentration, pipe flushing operation can be applied periodically [67]. This is, however, efficient in short time periods. To better control the lead pipe corrosion, there are several methods in use: pH/alkalinity adjustment, adding corrosion inhibitors (orthophosphate, orthophosphate/polyphosphate mixture), softening with Ca(OH)_2_ [68,69]. In the case of corrosion inhibition using orthophosphate, amorphous solid phase (containing mainly Pb, Al, P, and Fe) is formed. This layer is not very stable and can be easily detached when disturbances in flowing conditions take place [69]. 

### 2.2. Cement

Cement is frequently used for the manufacturing of pipes or as a protective layer on the inner surface of an iron or steel pipe. Cement is used as a coating to prevent the corrosion of pipe metallic surfaces because of the high pH of the cement lining. In this way, the isolation of metallic pipe from water is achieved and an alkaline environment near the pipe walls is created, which prevents corrosion. The protective layer can be applied during the manufacturing process of the pipes (prefabricated coated pipes) or during the renovation of corroded water pipes (manually coated pipes). Concrete pipes are cement-based pipes made by mixing cement (limestone), water, sand, and additives, which prevents the concrete pipe from cracking. 

Concrete pipes are among the oldest pipe systems to be employed for water transportation [70]. 

The types of cement allowed to be used in manufacturing the pipes for drinking pipes are Ordinary Portland Cement (OPC), High Alumina Cement (HA cement), Blast Furnace Slag Cement (BFS cement), Fly Ash Cement (FA cement), and Sulfate-Resistant Portland Cement (SR cement) [71]. 

**Figure 2:**
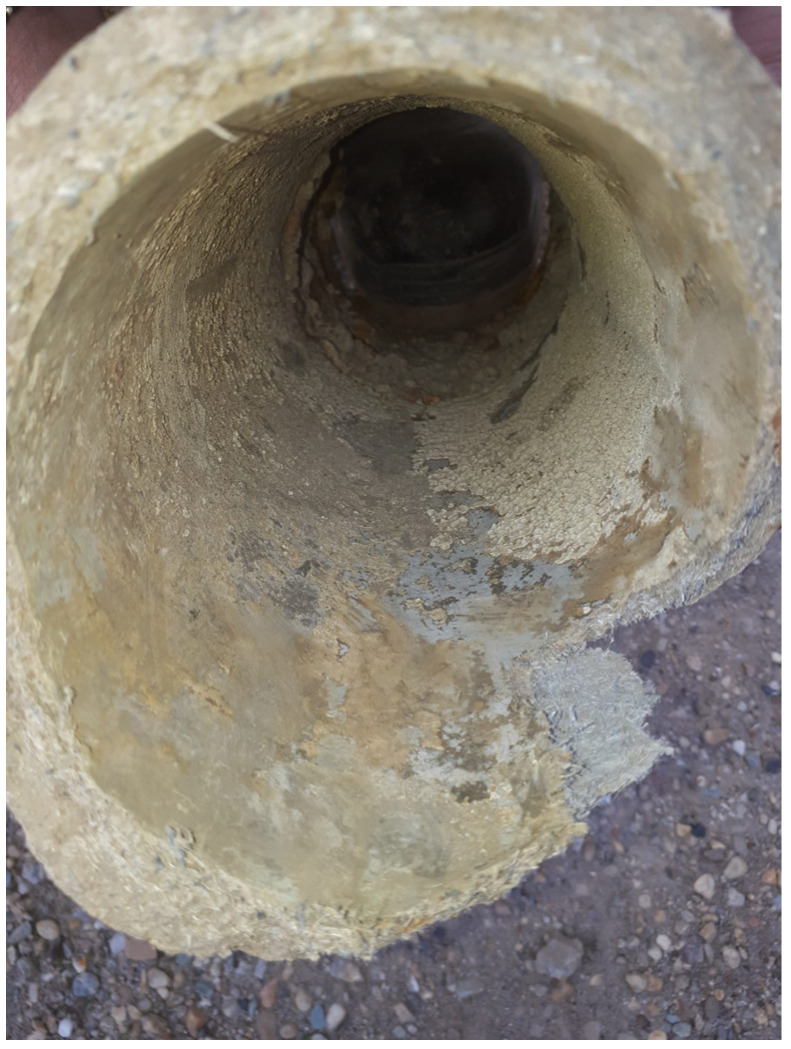
AC pipe replaced after about 50 years in service (courtesy of Ecoaqua S.A. Călăraşi, Romania).

Cement-manufactured pipes were usually used in combination with asbestos, which provides tensile strength and makes the pipes resistant to thermal and chemical breakdown. However, in the late 1980s, concerns were raised in New York regarding the use asbestos–cement pipes (AC) because the asbestos fibers were detaching from the inner pipe surface and migrating to drinking water [72,73]. Figure 2 shows the state of an AC pipe replaced from DWDS of the town of Călăraşi, Romania. The possibility of fibers contained in drinking water becoming airborne was studied. Results have evidenced an increased content of asbestos in the air of the houses being supplied with drinking water contaminated with asbestos fibers [73]. To this day, AC pipes are still in use for fresh water supply [74]. Mager et al. (2022) report the presence of asbestos fiber in drinking water originating from aging AC pipes in New Zeeland [75]. The pipe system was installed between 1930 and 1960, and to this date, was still releasing asbestos fibers: 10 μm long, with an average concentration of 0.9 million fibers per liter (MFL) and short asbestos fibers (0.5 μm) with an average concentration of 6.2 MFL. 

The analysis of a 56-year-old AC pipe evidenced surface corrosion because of calcium leaching from the wall [74]. Seasonal variation of temperature was found to impact the calcium dissolution rate, which, in turn, impacts the release of asbestos fibers: Lower water temperature increased the calcium leaching, which accelerates the detachment of asbestos fibers.

Cement is composed mainly of CaO, Al_2_O_3_, SiO_2_, Fe_2_O_3_, and gypsum in various ratios [76,77,78]. In addition to the characteristics of running water, the durability of asbestos–cement pipes is also directly related to the free lime content, Ca(OH)_2_ [79].

Concrete and cement pipes are mainly affected by the leaching process. Contaminants are released from the cement layer immediately after the installation of new pipes or after the rehabilitation of old pipes. The degree of water contamination is strongly related to the composition of the cement and is more accentuated at the beginning of the pipeline operation [71,80,81,82]. The leaching process means the dissolution of some compounds of the pipe walls. The dissolution takes place in the pores of the wall and assumes several steps:

(i) Internal diffusion of liquid water through the porous wall;
(ii) Dissolution of the compounds;
(iii) Diffusion of the dissolved compounds through the pores at the surface of the pipe;
(iv) External diffusion of the dissolved compounds at the wall pipe into the main water stream. The permeability of the material controls how much water diffuses through the pores of the material. The leaching rate depends on whether the pipe has just started to be operated, the permeability of the pipe material, the softness of drinking water, and the solubility of the leached compound. Soft water is water relatively free of dissolved ions and has a higher capacity for dissolution compared with more saturated water in minerals.

Cement is a source of toxic elements that can be of natural origin or introduced into the cement composition during the manufacturing process. The cement industry has modified its production methodology to include the valorization of several types of waste materials (meat and bone meal, waste tires, solidified sewage sludge) as an alternative fuel in cement kilns [83,84,85,86]. Waste tires are the most common sources of antimony, cadmium, chromium, cobalt, lead, titanium, and zinc [83]. Solidified sewage sludge is a source of cadmium, chromium, zinc, vanadium, and cobalt in varying concentrations [84]. No toxic heavy metals are leached from concrete manufactured using meat and bone meal waste [85].

Experimental studies conducted on cement-coated iron and steel pipes showed that calcium, aluminum, chromium, lead, and cadmium are the main ions that are more susceptible to being leached from the cement surface [81,87]. Calcium hydroxide was found to be the most soluble compound in cement [88]. For cement-coated iron and steel pipes, the leaching of aluminum was found to be much more intensive from manually prepared coating than from prefabricated cement coating. The type of cement used for the experiments was BFS cement in the case of prefabricated pipe and OPC for the manually coated pipe. The cumulative quantity of aluminum leached was about 160 mg/m^2^ for manually coated pipe versus 0.5 mg/m^2^ for prefabricated pipe, 100 days after starting the pipe’s operation [81]. Accelerated studies consisting of exposing samples of concrete to deionized water or acid solution for 64 days were made to see which type of cement (HA, BFS, or FA) is more suitable for use in contact with drinking water. High amounts of calcium and aluminum were leached from HA cement, whilst the smallest total amount of calcium and aluminum was measured for BFS cement [71]. For the countries in the European Union, the limit of aluminum in drinking water is set to 200 μg/L, according to the Directive (EU) 2020/2184 [1]. 

Comparison between CEM I types (OPC) made by two different manufacturers showed similar behavior. For both types of cement, the leaching of aluminum and calcium was very intensive in the initial contact with drinking water and decreased over time. Aluminum leaching was still present after a 64-day investigation period, while calcium was leached only up to 4 days after the first contact with drinking water. Leaching of chromium was similar for both types of cement CEM I and disappeared after 7 days. Lead leaching was not detectable after the fourth day for both types of cement. The main disadvantage when using CEM I type cement was exceeding the aluminum maximum concentration in drinking water for several days after the pipe was put in use [82]. 

Another parameter that affects the leaching of dangerous compounds from cement coating is the dose of disinfectant present in drinking water. The disinfectant is added to ensure a reduced concentration of microorganisms in water transported from the treatment plant to the consumer. Resistance to chloride ions of concrete-based pipes was extensively evaluated [89,90,91,92,93].

Adding sodium hypochlorite to water does not increase the water pH at contact with cement lining. However, when comparing results obtained from tests on water with or without disinfectant, the presence of disinfectant does considerably increase the leaching of different compounds (such as calcium, aluminum, chromium, and lead). The lowest tested sodium hypochlorite dose was the most aggressive, causing the highest rate for calcium, aluminum, and chromium leaching. This indicates that the effect of disinfectant on the leaching rate is indirectly proportional to the disinfectant concentration [87].

Experiments with cements having different compositions have shown that there is a dependence between the chemical composition of the cements and the type and concentrations of leached elements into water [82].

Another aspect observed after the installation of a new cement-coated pipeline was the significant increase in water alkalinity [94]. One day after the operation started, the water pH increased to 11 (the same pH value that was measured for the water inside the pores of fresh concrete material). The experiments were performed with still water (non-carbonated water). The pH values were above 8.5 even after 35 days of water flow, although the pH was continuously decreasing according to a logarithmic path.

To improve the resistance of cement pipes, PVC (polyvinyl chloride) tubes can be used as the inner layer [95]. There are, however, some inconveniences when using cement-based pipes with embedded polymer pipe. The parameters that were studied were pipe diameter, curing age, temperature, and resistance to chloride ions. These factors change with increasing the pipe diameter. Between the two pipes, a weak zone is formed, and this zone increases with the pipe diameter. The weak zone is characterized by reduced compressive strength and a higher diffusion coefficient of chloride ions [96]. 

Despite the alarm signals regarding the use of asbestos-based pipes, they are still used worldwide. The European Commission banned the manufacture and sale of asbestos-containing products through Directive 1999/77/EC, which entered into force on 1 January 2005. However, the directive states that asbestos-containing products can remain in service until they are disposed of or reach their end of life [97]. Despite the number of studies linking gastrointestinal cancer with constant ingestion of asbestos, Canada, USA, New Zeeland, and Australia do not ban the use of asbestos-containing pipes. There are large DWDS containing asbestos that are slowly replaced [74,75,98,99,100,101].

### 2.3. Copper

Comparison with steel [102], copper and copper alloys are corrosion scale-forming materials [103,104,105]. This means that the corrosion by-products accumulate on the interior of the pipe. It was found that pure water does not contribute significantly to the corrosion of copper pipes [106]. Generally, there are three types of scale in pipes:

(a) Compounds that crystallize directly onto tube surfaces from the flowing water (this is the case of calcium and magnesium carbonates that distribute uniformly on the inner surface of the pipe);
(b) Scale consisting of inorganic materials precipitated elsewhere and transported by the flowing water;
(c) Scale formed by the corrosion products (characteristic for unlined iron cast pipes, copper pipes); the corrosion scale is a hard mineral consisting of densely distributed corrosion tubercles.

At the beginning of their operation, the copper pipes are covered by a protective layer of cuprous oxide. 

The difference in the behavior of stainless steel and copper and copper alloy materials resides in the fact that the copper corrosion is much faster, and the concentration of copper ions in the water near the pipe wall rises, so the precipitation and crystallization of corrosion products take place. Stainless steel also forms a passivation film, but the corrosion of this film is so slow that the corrosion products do not accumulate at the pipe wall. Scale build-up is a process that takes months or even years. It is difficult to predict how the existing corrosion scale will behave when water composition changes or seasonal changes take place [107].

Copper corrosion in the presence of soft water at room temperature and flowing water is initiated by the breakdown of the thin metallic oxide layer. The cuprous oxide crystals that are separated from the layer migrate to form mounds at grain boundaries. Spherical pits are created in this way. The presence of chloride ions used for water disinfection accelerates the breakdown of the oxide layer [108]. 

The main factors that were found to affect the copper corrosion and later to cause the leaching of the corrosion products in drinking water are:

- pH [106,109];
- temperature [110];
- total organic carbon [110];
- dissolved inorganic carbon (carbonates) [106,107];
- chloride ions or other disinfectant substances [110,111];
- corrosion inhibitors: sulfate, polyphosfate, and orthophosfate [106,109,112,113];
- existence of microorganisms in biofilm [114,115,116,117];

The components of the scale formed by the corrosion products were products of Cu(I): cuprite (Cu_2_O) and copper (I) hydroxide (CuOH) and products of Cu(II): CuO, copper (II) hydroxide (Cu(OH)_2_), and malachite (Cu_2_CO_3_(OH)_2_) [106]. 

By increasing the velocity of water flowing in the pipe, the copper release also increases [118]. 

Experiments with a lead-free laboratory system of pipes (consisting of copper pipes, stainless-steel taps, and brass fittings) evidenced elevated levels of lead leaching [119]. The samples were analyzed after 475 days of alternative flowing/stagnation periods. Concentrations of 10 μg lead·L ^−1^ were measured for five months after 475 days, the source of lead was identified as a lead-free brass fitting.

### 2.4. Iron

The iron pipes used for DWDS are cast iron pipe, galvanized iron pipe, unlined cast iron pipe, and lined ductile iron pipe.

Corrosion of metallic iron means its dissolution from the metallic pipe as Fe^2+^ simultaneously with the reducing of oxidizing agents present in water: O_2_, H^+^, SO_4_^2−^, NO^2−^, NO^3−^, CO_2_; ferrous iron can be further oxidized to ferric iron. This ends up creating cracking and pitting iron corrosion. The primary parameters that affect the iron pipe stability are related to the flowing water characteristics: alkalinity, pH, presence of chloride and sulfate ions [120,121,122,123,124,125,126]. Orthophosphates added to the flowing water act as corrosion inhibitors by forming insoluble phosphates that create a coating protective layer [127]. 

**Figure 3:**
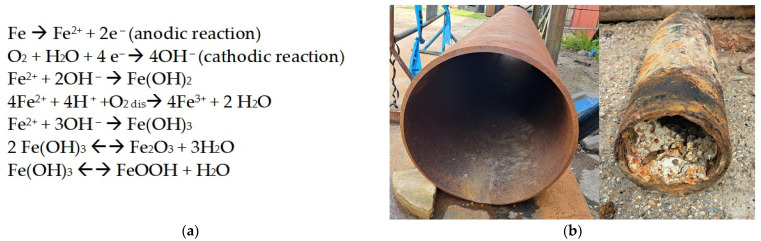
(**a**) Schematic representation of corrosion reactions; (**b**) new iron pipe and old iron pipes (courtesy of Ecoaqua S.A. Călăraşi, Romania).

The metallic surface (iron, steel, etc.) acts as the anode, while the water near the pipe surface (containing oxidants: oxygen, chlorine, microbial metabolites) participates as the cathode (Figure 3).

The solid corrosion products that build up thick corrosion scale are responsible for pipe blockage, leakage, and red water formation [128,129]. Corrosion scales formed on the surface of pipes consist mainly of lepidocrocite (γ-FeOOH), magnetite (Fe_3_O_4_), goethite (α-FeOOH), hematite (Fe_2_O_3_), and ferrihydrite [130,131,132]. All these minerals have strong affinities to adsorb heavy metals (lead > vanadium > chromium > copper > arsenic > zinc > cadmium > nickel > uranium) [130]. Another source of heavy metals are the pipes themselves. The iron pipes can contain other metals in small amounts. As the iron pipe starts to corrode, these metals are released from the pipe wall and incorporated into the corrosion by-products. Red water is formed under stagnant conditions due to oxygen depletion in water by iron, existing Fe(III) is reduced to Fe(II), followed by the dissolution of corrosion by-products [123,133,134]. Measurements of iron corrosion potential in flowing and stagnant water confirmed the mechanism proposed by Kuch (1988) [133]. Experiments carried out with dissolved oxygen evidenced that the increase in iron corrosion potential during stagnation periods is associated with the consumption of dissolved oxygen by the iron [123]. The corrosion rate of cast iron in drinking water distribution pipes decreases gradually over time [135].

In the soil, ductile iron pipes can be protected from external corrosion by polyethylene encasement [136].

The metal accumulation in corrosion scale represents a threat to the population’s health. A maximum accumulation rate of 3.94 mg vanadium/g of scale and 3.90 mg arsenic/g of scale, respectively, was found by He et al. (2021) on scales from cast iron pipe (20 years operation time) [132]. The accumulation rate depends on various factors, such as pH and temperature. The scale is mainly composed of magnetite and goethite. Although the scale layer is thinner on the steel pipes, it was also reported that the scale deposited in the steel pipes also accumulates vanadium. The adsorption capacity in the different layers of the corrosion scale decreases in the following order: surface layer > porous core layer > hard shell-like layer [137]. Calculations on iron corrosion scale showed that it is sufficient to dissolve 0.0027% of a 0.1 cm thick corrosion scale (for a 1 m segment length and 3 cm diameter) to reach the limit for vanadium in drinking water, which is 15 μ/L [138].

Chloride and sulfate anions diminish arsenic accumulation, therefore, increasing their content will reduce arsenic accumulation on corrosion scales [132]. 

Another toxic heavy metal is chromium. The accumulation and release of chromium (III) and (VI) from iron corrosion scales was investigated [139]. The outer layer of the scale accumulates less chromium and releases more. During the Cr^6+^ accumulation process, part of the Cr^6+^ is reduced to Cr^3+^ by the existing Fe^2+^, which is a beneficial process since the toxicity of Cr^3+^ is reduced. 

Comparing with the stainless steel scale, which contains chromium from the corrosion products, the iron scale accumulates chromium from the water flowing in the pipe or from the inner linings of the pipe. The chromium content in scales of stainless-steel pipes is higher than the chromium content of iron corrosion scales [102]. 

The accumulated heavy metals can be released in some conditions. Vanadium release is increased by low pH, high temperatures, and high sulfate ion concentration [138]. Chromium release is affected by several factors in a decreasing order: pH > temperature > chloride ion concentration > sulfate ion concentration [139].

Manganese also can accumulate in iron pipe scales. Although not as dangerous as vanadium, chromium, and arsenic, its concentration in drinking water is monitored and limited to 0.05 mg/L [1]. The manganese release is enhanced in stagnant water. The factors influencing the manganese release are pH, alkalinity, sulfate ion concentrations, and the presence of disinfectants [140]. Like in the case of vanadium release, lower pH, lower alkalinity, higher temperature, and higher sulfate concentration increase the manganese release from the scales. Under the same contact time and disinfectant dose, the disinfectant inhibitory effect on the manganese release follows the order: ClO_2_ > NaClO > NH_2_Cl. Two indicators, alkalinity and sulfate ion concentration, have a strong effect on manganese release. They could be monitored to control the manganese release from iron scales. 

**Figure 4:**
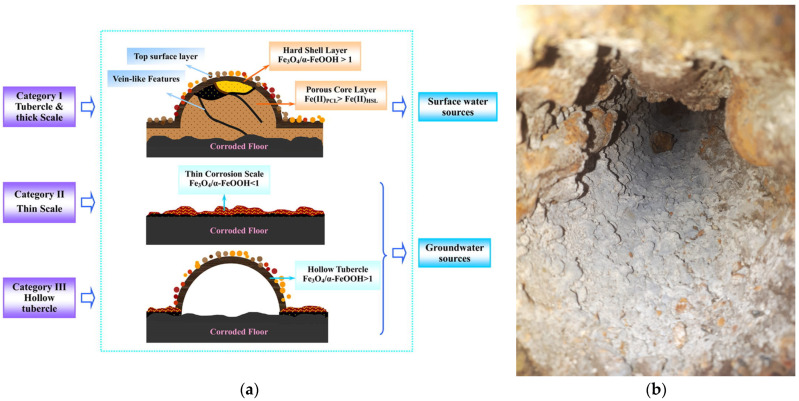
Types of iron corrosion scale (**a**) schematical representation [141]; (**b**) appearance of corrosion scale on cast iron pipe (courtesy of Ecoaqua S.A. Călăraşi, Romania).

Similarly to copper pipes, iron scale is composed of layers (Figure 4) [141]:

(a) Floor layer made of carbonates;
(b) Thin corrosion scale;
(c) Hollow tubercle.

**Figure 5:**
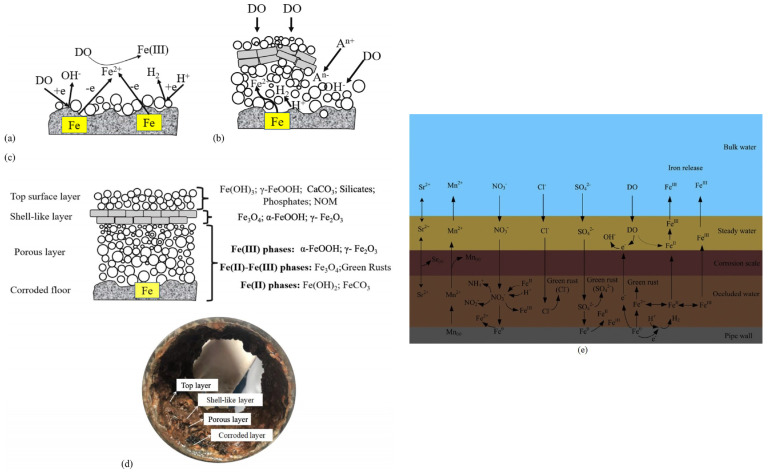
Stages of corrosion scale formation: (**a**) initial stage; (**b**) intermediate stage (note: A^n−^ represent ions); (**c**) stable stage; (**d**) image of actual iron pipe corrosion scale from DWDSs in Hangzhou city of China [142]; (**e**) Migration and transformation of ions among bulk, steady, and occluded water [153].

Over time, the corrosion scale formed on iron pipes develops a multi-layer structure consisting of the corroded floor, which is the pipe’s inner wall, porous layer, stable shell layer, and top layer (Figure 5). The shell layer gives resistance to further corrosion process by blocking the access of oxidizing compounds, being mainly composed of compact α-goetite and magnetite: α FeOOH (more than 50%), followed by calcium carbonate (about 20%) and Fe_3_O_4_ (below 10%) [142]. For water pipes that do not undergo CaCO_3_ deposition, the percentage of α FeOOH and Fe_3_O_4_ are almost of the same order of magnitude (about 40%), being followed by β-FeOOH [141]. The differences in the composition of the scales can be attributed to the different drinking water composition (the presence or the absence of calcium carbonate) and the pretreatment type of the pipe surface (presence or absence of iron carbonate, decreased content of Fe_3_O_4_ and β-FeOOH) [141,143].

The water characteristics’ and disinfectants’ influence on iron corrosion in a ductile iron pipe were studied in accelerated laboratory tests using increased concentrations of disinfectant [144]. Sodium hypochlorite, like liquid chlorine, enhances iron corrosion by oxidizing ferrous to ferric ions. The effect of NaClO on the presence of calcium ions is different from the chlorine effect, NaClO is responsible for calcium carbonate accumulation on the inner pipe wall in waters with high alkalinity and hardness. NaClO not only contributes to pipe corrosion but also intensifies the CaCO_3_ deposition over time. 

Nitrogenous disinfection by-products (N-DBPs) are known to be more dangerous to human health and the environment than carbonaceous DBPs [145,146,147,148]. Increased content of N-DBPs (haloacetamides, halonitromethanes, and haloacetonitriles) and iron particles was found in the effluent of corroded iron cast pipes [149]. It was found that detaching iron particles from the corrosion scale are covered by the biofilm. This provides a good surface for chlorine adsorption and N-DBPs formation. 

The iron release rate from corrosion scale was found to be dependent on pH, hardness, nitrate ion content, Larson Ratio (ratio of the concentration of chloride and sulfate ions to the concentration of bicarbonate and carbonate ions), and dissolved oxygen [134]. 

The formation of siderite (FeCO_3_) was also found to play an important role in iron corrosion [122,150]. Scales formed on different pipes (PVC; lined ductile iron, LDI; unlined cast iron, UCI; galvanized steel, GS) after 1 year of use, with water having different characteristics, were analyzed [122]. The order of total iron release decreases in the order: unlined cast iron > galvanized steel ≥ lined ductile iron > PVC. The main corrosion by-products on unlined cast iron pipe were: FeCO_3_, α-FeOOH, β-FeOOH, γ-Fe_2_O_3_, Fe_3_O_4_, while on galvanized steel, FeCO_3_ was replaced by significant amounts of zinc oxide. Lined ductile iron and PVC had low deposits of iron containing corrosion by-products. 

Higher amounts of zinc in drinking water were for galvanized iron pipe, GI, reported also in the study of Li et al. (2016), when comparing the behavior of UCI and GI pipe [151].

The scale deposited on iron-based pipes provides a favorable environment for microinvertebrates (Asellus aquaticus) to develop and live in large numbers. On the contrary, on plastic pipes, there were only isolated specimens of Asellus aquaticus [152]. 

The effect of sulfate ions was studied for old cast iron distribution pipes [125]. Red water occurred for the pipes usually fed with groundwater, while no coloration was noticeable for pipes supplied with surface water. The difference arises from the scale composition: (i) thin and less stable for the groundwater-supplied pipes containing mainly higher proportion β-FeOOH, FeCO_3_, and green rust; (ii) thick and more stable for surface water-supplied pipes, having a high content of stable Fe_3_O_4_. The water sulfate content also influences the bacterial communities living on the scale surface. While the bacterial community did not vary much for pipes transporting treated surface water, in the case of pipes transporting groundwater, changes in water with sulfate increase the content of sulfur-oxidizing bacteria (SOB), sulfate-reducing bacteria (SRB), and iron-oxidizing bacteria (IOB). The authors did not study the sulfate effect on the release of dangerous heavy metals, such as vanadium and chromium. 

During the corrosion process, water can be trapped in the scale (occluded water). The contaminants migrate from the occluded water to the bulk water or to the stagnant water, therefore, occluded water is another source of drinking water contaminants. This water is acidic (e.g., pH = 5.61 in flowing conditions) and contains high amounts of iron, manganese, chloride, sulfate, and nitrate [153]. In stagnant conditions, the occluded water becomes more acidic (pH = 4.48 in stagnant conditions) and concentrated in the compounds mentioned above, except for nitrate. It was found that the tubercles have an internal porosity of 40 to 54% [154]. The interior of the tubercles is filled with water [66]. The tubercles have poor mechanical strength, and the volume of the water inside is relatively small. Attempts to analyze the chemical composition of occluded water were hindered by the difficulty of water sampling from tubercles. Tong et al. (2015) developed a method consisting of drilling small holes inside tubercles [153]. The source of manganese and iron inside the tubercle is an anodic reaction that contributes to metal release from the wall pipe. Because the tubercle wall acts as a barrier to metal migration, the water inside the tubercle concentrates in manganese and iron [155]. When the hydraulic conditions change (passing from flowing to stagnant conditions), the composition of occluded water changes [153]. It was found that the concentration of chromium and copper in the bulk water is also dependent on the hydraulic regime. In Figure 5e, the migration of different ions among the layers of the corrosion scale is schematically represented. For a better understanding, the occluded water is represented with brown color (the samples extracted from tubercles were black or brown, with suspended corrosion products), while steady water is represented with light brown because it contains fewer corrosion products. 

### 2.5. Steel

Stainless-steel pipes are extremely resistant to corrosion. However, under long-term operation, they become susceptible to pitting corrosion, which forms thin layers of corrosion scales. 

**Figure 6:**
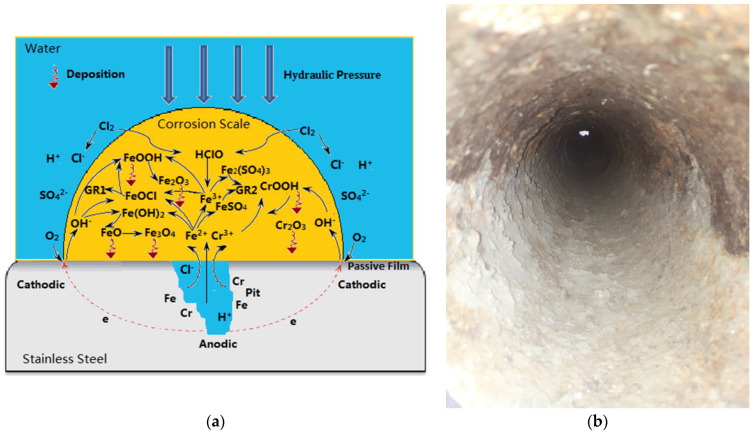
Steel corrosion: (**a**) schematic representation of formation mechanism of the stainless–steel corrosion scale [102]; (**b**) appearance of corrosion scale on steel pipe (courtesy of Ecoaqua S.A. Călăraşi, Romania).

It has been reported that stainless-steel corrosion scales contain a large amount of chromium compounds. The scale formation is initiated by pitting corrosion on the pipe surface, followed by the homogeneous deposition of iron and chromium corrosion compounds (Figure 6) [80]. The insoluble corrosion products are α-FeOOH, α-Fe_2_O_3_, γ-FeOOH, γ-Fe_2_O_3_, Fe_3_O_4_, FeCO_3_, Cr_2_O_3_, CrOOH, and possibly FeSO_4_. 

Physico-chemical characterization of pipe scales can be made by SEM, XRF, XRD, and XPS [156,157]. 

**Figure 7:**
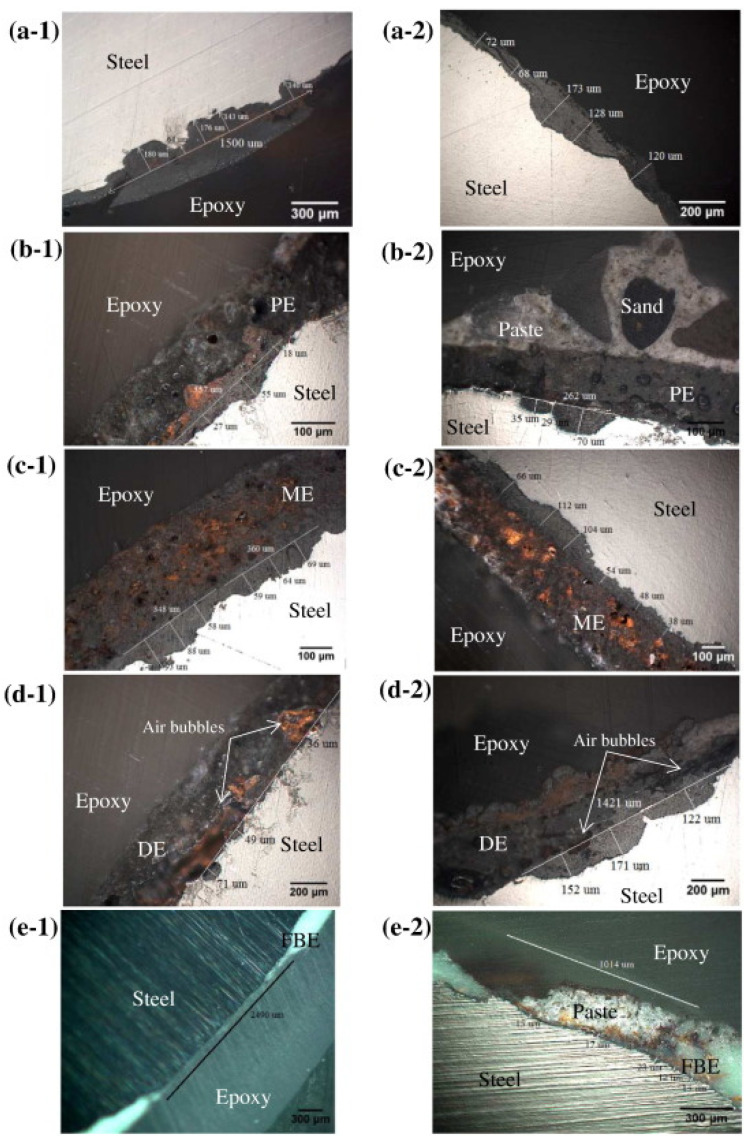
Cross-sectional view of corrosion pits on (**a**) uncoated, (**b**) PE-pure enamel coating, (**c**) ME-mixed enamel coating, (**d**) DE-double enamel coating, and (**e**) FBE-fusion bonded epoxy coated steel bars; (1)—initially; (2)—after 244 days of test [158].

The presence of disinfectant products enhances the corrosion of stainless-steel pipe [102,158]. Steel pipe corrosion in the presence of chloride ions is a multi-step process that depends, besides the characteristics of water, also on the presence or absence of a coating protective layer. For uncoated steel pipes, three steps were evidenced during chloride-induced corrosion: (a) diffusion of chloride ions through the mortar/cement layer; (b) pitting corrosion by which cavities are produced in the steel layer; (c) transition from pitting corrosion to uniform corrosion as more and more chloride ions arrive at the surface of the steel layer. Figure 7 shows comparative cross-sections of steel bars with different enamel or polymer protective layers [158]. The enamel protective layer did not postpone the initiation of the corrosion process; only the epoxy protective layer delayed the pitting corrosion. 

Under stagnant water, the release of heavy metals from a galvanized steel pipe does not exceed the maximum allowable concentration limits for stagnation time below 8 h. The heavy metal concentrations decreased in the following order: Mn > Fe > Zn > Pb [156]. 

It was found that pipe corrosion is reduced in flowing conditions compared to stagnant conditions: In low water flow or stagnant conditions, the corrosion rate is 0.29 mm/year and decreases to 0.18 mm/year for water flow rate above 0.15 m/s [157]. 

### 2.6. Polymer-Based Pipes

Polymeric pipes used in DWDS include PE (polyethylene), PEX (cross-linked polyethylene), LDPE (low-density polyethylene), HD-PE (high-density polyethylene), PVC (polyvinyl chloride), PVC-U (unplasticized polyvinyl chloride), and Hi-PVC (high-impact polyvinyl chloride) pipes.

Polymeric pipes have some advantages compared to other pipes: They are lighter, which is important if they are used in buildings, and they do not form corrosion scale, etc. Among analyzed pipes (PVC-U, galvanized steel pipe, copper pipe, and cast-iron pipe), PVC-U pipes have the minimum value in terms of resource and energy consumption in residential buildings [159].

By using polymer-based pipes in DWDSs, the problems related to compounds containing heavy metals are reduced significantly. However, polymer-based pipes have a reduced mechanical strength and favor the formation of organoleptic compounds [160,161] and biofilm on the inner pipe surface [162]. To improve mechanical strength, polymer-based pipes are mostly used as inner pipes in cement- or metal-based pipes. 

Experiments have shown that defective polyethylene tubings (dissolution of polymer additives and oxidation of pipe surface during extrusion) can explain the formation of organoleptic substances (alkylphenols, aldehydes, and ketones). The release time of organoleptic compounds was found to exceed several months under low water flow rates [160]. 

It was shown experimentally over 16 weeks of investigation that the migration of organic compounds was elevated within the first weeks of use, followed by a lower and constant level [163]. The organic compounds that migrate from the pipe surface (polyethylene and cross-linked polyethylene) can be further transformed and degraded in bulk water [164]. These compounds can be degraded biotically or abiotically, but the degradation rate is low. However, this study did not include the influence of pre-existent biofilm on the pipes’ inner surface. It is expected that the presence of the biofilm would change the degradation rate. 

MTBE (methyl tert-butyl ether) was found to be leached from newly installed PEX pipes. In the first months, MTBE concentrations were above the recommended US EPA limits but decreased below this value after 5 months in service [165].

Reactions between water and the different materials of DWDS can result in alterations in water quality delivered to consumers. The DWDS consists of pipes of different materials that are replaced over time. The iron can dissolve itself from iron-based pipes and migrate and concentrate on inner surface of polymer-based pipes [166,167]. The study by Wang et al. (2019) evidenced that water quality parameters (e.g., contents of content, sulfate, bicarbonate, and humic acid, and water pH) affect the iron release from the polymer-based pipes’ inner surface [166]. Like iron, manganese can also be deposited from the flowing water on PVC pipes [168]. The behavior of PVC pipes and iron pipes is different regarding the deposition of manganese present in drinking water: The PVC pipe provides more favorable conditions for manganese adherence, but the layer formed is not stable and easily detaches.

PVC pipes release different traces of additives used in the manufacturing process (e.g., organotins or lead-containing additives). It was recommended that PVC should be rinsed before operation to eliminate the organotin traces [169]. It was found that lead additives leach from unplasticized PVC pipes under the action of UV radiation (a concentration of 0.8 mg/L was detected after 14 days of exposure) [170].

It was found that the formation of biofilm is accelerated when the water velocity increased. This is because when increasing the water velocity, the mass transfer of nutrients is intensified. Increased growth of biofilm with water velocity was reported for both copper and plastic pipes. However, contrary to plastic pipes, in the case of copper pipes, the increase in water flow rate results in an immediate increase of bacterial numbers and copper concentration in water caused by deposit detachment [171]. 

The major migration component from HDPE pipes was 2,4-di-tert-butyl-phenol (2,4-DTBP), which is a degradation product of polymer additive (antioxidants). To evaluate the odor level, TON (threshold odor number) was used. Among tested pipes (HDPE, PEX, and PVC), PVC pipe showed no significant odor [172]. When comparing newly installed PE and HDPE pipes, HDPE pipe released smaller quantities of antioxidants and their degradation products in the drinking water distribution system [173]. Retention of lead from drinking water on biofilm grown for three months was evaluated for PEX, HDPE, and copper pipes. The lead accumulation experiments were performed for five days. Results showed that PEX pipes developed a thicker layer of biofilm compared to HDPE and copper pipes. The lead level accumulated on the pipe wall was correlated with the presence of biofilm [174].

Although polymeric pipes are not susceptible to corrosion by scale formation, their mechanical structure can, however, be impacted by oxidation following the use of disinfectants. 

The effect of two common disinfectants (chlorine dioxide, ClO_2_, and sodium hypochlorite, NaOCl) on polymer-based pipes was studied in accelerated aging experiments. The evaluated pipes were HDPE, LDPE, PVC–U, and Hi-PVC pipes. The pipes have a lower resistance to oxidation in the presence of ClO_2_ than in the presence of NaOCl. This can be explained by the fact that ClO_2_ generates free radicals that contribute to C–C chain scissions [175]. 

New types of pipes combine the use of conventional pipes, such as ductile iron pipes or steel pipes, and polymer coatings. Steel-reinforced high-density polyethylene (SRHDPE) is an example of pipe that merges the mechanical properties of steel with the corrosion resistance of polymers [176]. However, this type of pipes is preferably used in the oil industry or in wastewater transport [177]. Their behavior over time is not known for the moment.

The main substances that can be leached from different components of DWDS are summarized in Table 2.

## 3. Biofilm Formation on Inner Pipe Wall

Biofilms formed on the inner walls of water distribution pipes are responsible for several types of problems, such as the deterioration of water quality, corrosion of metallic pipe walls, intensifying the leaching processes in concrete-based pipes, proliferation of pathogens, etc. The negative action of microorganisms in biofilm can be intensified by the existence of scale on the inner pipe walls. 

It is known that the number and diversity of microorganisms in the film is higher than that in flowing water [178].

The biofilm and the scale formed on the pipe walls can exfoliate, releasing heavy metals and pathogens into drinking water [171,179,180]. The biofilm can contain microorganisms, such as *Pseudomonas aeruginosa* and *Legionella pneumophila*, that create health-related issues [181,182,183]. Moreover, the biofilm can be responsible for the deterioration of the taste and odor of drinking water [184].

The source of microorganisms can be the water itself or cracks in the pipe distribution system that are not remediated.

The biofilm produced by microorganisms present in the flowing water adheres to the surface of the inner pipes and is formed by different constituents of extrapolymeric substances: carbohydrates, lipids, proteins, uronic acids [131], etc. 

The steps of biofilm formation include the following successional steps: (i) microorganisms from the bulk water attach to the pipe surface; (ii) microorganisms start to develop, releasing extracellular polymeric substances (EPS); (iii) modify the surroundings to exclude or enhance the development of other microorganisms [2,185].

The factors influencing the growth of the biofilm include the water source [186,187,188], availability of the nutrients [189], disinfectant concentration [185,190,191], water flowing regime [192,193], and the pipe material [162,185,190,194,195,196,197,198,199,200,201].

There are several methods for determining the microbial growth potential of materials:

- Mean Oxygen Difference (MDOD) consists in the determination of additional oxygen consumption in the presence of the tested material [202]
- Slime Production (SP) test measures the volume of slime on the surface of the tested material. If SP value exceeds 0.1 mL/800 cm^2^, the material is considered unsuitable to be used in DWDS [203]
- Biomass Production Potential (BPP) Test uses Adenosinetriphosphate (ATP) quantification as a measure of active biomass [204].

The main microorganisms involved in the pipe corrosion are sulfate-reducing bacteria (SRB) [205,206], nitrate-reducing bacteria (NRB) [207,208], acid-producing bacteria (APB) [209], and metal-oxidizing bacteria (MOB) [210,211]. A particularity of the biofilm effect on pipe corrosion is its non-uniform composition, inducing a non-equal distribution of the corrosion process on the pipe surface [210]. The BART test can be used to identify and count different bacteria in DWDS. It provides multiple environments in the presence of which specific bacteria are activated [212]. IOB isolated from different pipes scale samples was grown on a Winogradsky nutrient medium and determined by the plate-counting method [131,210]. Bacteria in biofilm are characterized using several tests: bacterial growth on R2A broth to analyse cell surface hydrophobicity; determination of zeta potential; assessment of cell motility that included swimming, swarming, twitching, and autoaggregation [205]. Besides bacteria, the biofilm composition can also include viruses [213]. The proportion of both bacteria and viruses in water increases during stagnation periods. 

**Figure 8:**
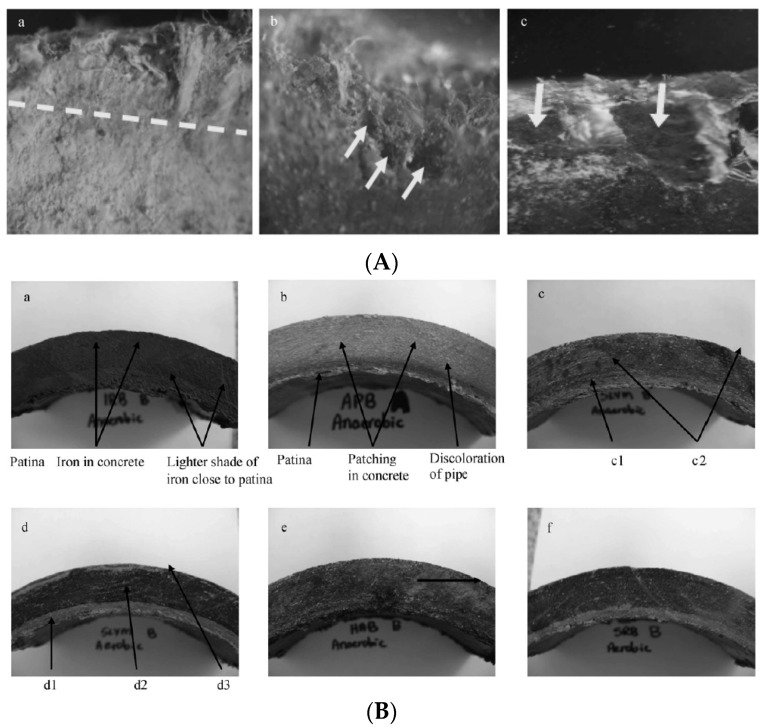
(**A**) (**a**) Inside wall of AC pipe is coated with a layer of patina 3–4 mm depth; (**b**) patina is thick fibrous films that are composed of high fibrous asbestos and some biomass forming gelatinous-like materials; (**c**) patina tends to have yellow to brown colorations implying the presence of some iron. (**B**) AC segments after 10 days cultivation under different conditions. (**a**) IRB in anaerobic conditions; (**b**) APB in anaerobic conditions; (**c**) SLYM in anaerobic conditions, c1: layer of discoloration immediately behind the patina 4–6 mm, c2: discolored patches within the concrete; (**d**) SLYM in aerobic conditions, d1: patina, d2: main body, d3: outer layer; (**e**) HAB in anaerobic conditions, Methylene Blue was found on patina and the layer immediately behind patina; arrow indicates a weathering spot on the segment; (**f**) SRB in aerobic conditions (IRB—iron-reducing bacteria; APB—acid-producing bacteria; SLYM—slime-forming bacteria; HAB—heterotrophic aerobic bacteria; SRB—sulfate-reducing bacteria) [212].

Concrete was found to be susceptible to degradation under the action of microorganisms [214,215]. Biofilm formation has also been reported in the case of AC pipes. Microorganisms can bioaccumulate to form a layer of 2 to 5 mm thickness, colored in yellow, orange, brown, or black depending on the metallic cations that have been extracted from the AC pipe wall [212,216]. In this film, several layers of microorganisms can be distinguished (Figure 8). By performing experiments with microorganisms cultivated on new AC pipes, the steps of pipe degradation and consequently, pipe failure have been evidenced: (i) the fatty acids generated by bacterial growth, especially under anaerobic conditions, decrease the pH locally; (ii) the concrete structure is deteriorated by leaching the free lime, leaving the asbestos fibers as a woven matrix inside the pipe wall; (iii) the path inside the fiber matrix will allow more development of the bacterial film growth; (iv) the asbestos structure weakens, detaches, and is released in drinking water; (v) the structural strength of the AC pipe is reduced.

Microbiologically induced corrosion (MIC) affects metallic pipes. Worldwide, there is a major concern related to the economic impact generated by maintenance of the water distribution system. Microbial-induced corrosion, together with scale accumulation, may damage the pipeline, resulting in pipe leakage or bursting. Biofilm formation can contribute to the acceleration of metallic pipe corrosion [114,115,116,117,217]. Pitting corrosion starts at the interface biofilm–metallic pipe because the water pH is modified by the release of metabolic degradation compounds [117].

**Figure 9:**
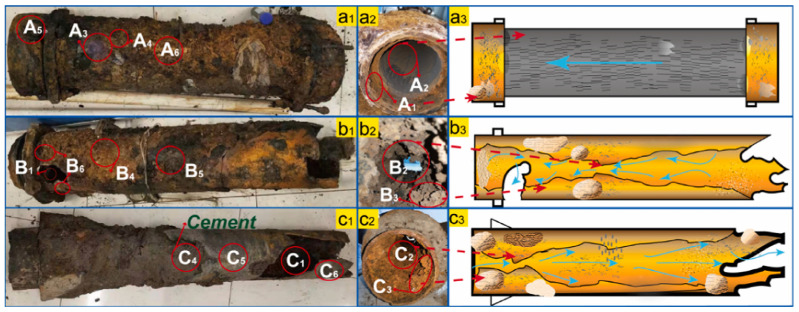
Cast iron pipes: corrosion scale image of pipeline inner and outer walls. (**a_1_**,**b_1_**,**c_1_**) represent the appearance of lined pipe A, unlined pipe B, and unlined pipe C, respectively; (**a_2_**,**b_2_**,**c_2_**) represent the internal corrosion of lined pipe A, unlined pipe B, and unlined pipe C, respectively. (**a_3_**,**b_3_**,**c_3_**) corresponding to (**a_2_**,**b_2_**,**c_2_**) represent the internal pipe scale extraction location. The sample labels A_1_–A_6_, B_1_–B_6_, and C_1_–C_6_ represent sampling locations. Note: Inner wall scale: A_1_, A_2_, B_1_, B_2_, B_3_, C_1_, C_2_, and C_3_. Outer wall scale: A_3_, A_4_, A_5_, A_5_, B_4_, B_5_, B_6_, C_4_, C_5_, and C_6_ [217].

To be able to prevent pipe bursting, it is important to characterize the scale accumulated on the inner surface of the pipe and to obtain information about the various microorganism that colonize the biofilm. An extended study on scale taken from cast iron pipes that were in service for 22 to 26 years was carried out by Jia et al. (2022) [217]. The pipes were affected by severe corrosion, burst, and leakage (Figure 9). FTIR and XRD analysis of pipe samples evidenced the presence of γ-FeOOH and α-FeOOH in loose scales and of Fe_3_O_4_ in rigid scales. Moreover, late-stage corrosion products (Fe_3_O_4_, α-Fe_2_O_3_, γ-Fe_2_O_3_, and FeCO_3_) were observed in the severely corroded samples. Among the types of bacteria that are known to play an important role in the processes of iron migration (iron-oxidizing bacteria, iron-reducing bacteria, sulfur-oxidizing bacteria, sulfate-reducing bacteria, ammonia-producing bacteria, nitrifying bacteria), sulfate-reducing bacteria and ammonia-producing bacteria were predominant in the biofilm.

It was found that in iron scales, the microorganism community has a bigger influence on Fe_3_O_4_ formation than water chemical parameters. This means that in iron pipes distributing drinking water, the microbial community influences the capture of iron from unstable corrosion products and enhances the formation of more stable and compact scale mainly consisting of Fe_3_O_4_ [218,219].

Corrosion of steel pipes used in water distribution systems in the presence of microorganisms has been extensively studied [131,220,221,222]. MIC of carbon steel enhances the uniform and localized corrosion of pipe material [109]. Iron-oxidizing bacteria (IOB) are responsible for the corrosion of cast iron pipes and carbon steel pipes [222]. Iron-oxidizing bacteria (*Pseudomonas* sp. strain DASEWM2) determinate a higher corrosion rate of the pipe by: (i) forming wider cracks in the corrosion layer in the presence of extrapolymeric substances; (ii) the presence of corrosion products with the non-protective effect of surface [131].

Under stagnant water, the biofilm formed on corroded copper and stainless-steel pipes is favorable to the formation and growth of Legionella pneumophila [183].

An extended study on the influence of pipe material on the evolution of the microorganism community was performed for water distribution systems in East China [196]. The study included samples of biofilm material from different pipe materials: ductile cast iron pipe (DCIP), gray cast iron pipe (GCIP), galvanized steel pipe (GSP), stainless-steel-clad pipe (SSCP), and polyvinyl chloride (PVC). Results showed that the biomass grows faster on iron-based pipes (DCIP and GCIP). The highest diversity of bacterial species is also found on iron-based pipes. The total iron content in the biofilm samples influences the distribution of bacterial communities. The iron concentration in the flowing water is higher in the PVC pipes than in the iron-based pipes, which suggests that iron accumulates in the biofilm. Analyzed samples of biofilm showed that iron and manganese accumulate in high concentrations in the biofilm formed on iron-based pipes. 

Comparison among four pipe materials (steel, copper, stainless steel, and polyvinyl chloride) showed that bacterial communities are more developed on steel and copper pipes [223]. These pipes form important corrosion scales compared with stainless-steel and PVC pipes. Stainless steel has the lowest bacterial count at the end of the operation. However, steel pipe has the highest bacterial diversity among metallic pipes.

Nitrate was found to influence the bacterial communities and iron release from the corrosion scales on cast iron and stainless-steel pipes [207]. The increase in nitrate content promotes the growth of nitrate-reducing bacteria while decreasing the activity of iron-reducing bacteria. The percentage of goethite decreases, and the predominant form of iron in the corrosion scales becomes magnetite (Fe_3_O_4_), which is more stable compared with other forms of iron oxides. Therefore, iron release in drinking water decreases.

The biofilm presence significantly contributes to disinfectant concentration decay [191,224]. The pipe material influences the growth and distribution of microorganisms in the biofilm. The use of disinfectant 2,4,6-trichlorophenol (2,4,6-TCP) induced the formation of the 2,4,6-trichloroanisole (2,4,6-TCA), which is a source of odor in drinking water. Following this, the interaction between the biofilm and the disinfectant will be different from one material to another. Ductile iron and stainless-steel pipes produced much higher amounts of 2,4,6-TCA than PE pipe [184]. 

Comparison among three pipe materials (ductile iron, cement-lined stainless steel, and polyethylene) used in the same conditions of flow and water characteristics showed significant differences in bacterial film growth and community [162]. The difference in the bacterial communities means that in the presence of disinfectant, each pipe will generate other disinfection by-products. Ductile iron had the highest disinfection by-product formation potential. This is in accordance with the biofilm density and thickness measurements: ductile iron biofilm > stainless steel biofilm > polyethylene. The dominant bacteria percentage was similar for ductile iron and stainless-steel pipes.

Another study comparing pipes made of PVC-U (polyvinyl chloride-unplasticized), PE-HD (polyethylene-high density), and cast-iron pipes showed that on polymeric pipes, the microbiome consists mainly of *Proteobacteria*, while on cast iron pipe, it consists of *Nitrospirae*. The study included 7 years old pipes with biofilm complex composition and concluded that compared to cast iron pipe, polymer-based pipes offer a more suitable environment for pathogenic organism development [198]. In a chlorinated DWDS, a community consisting of bacteria *Pseudomonas*, *Massillia*, and *Sphingomonas* and the fungi *Acremonium* and *Neocosmopora* was detected in the biofilm formed on PE-HD [225].

Under the same operating conditions, PE–HD pipes were more susceptible towards biofilm development while concrete pipes were susceptible towards metal deposition (iron and manganese especially) [226].

Adherence of *Legionella pneumophila* and *Pseudomonas aeruginosa* on several types of pipes (ethylene-propylenediene-monomer rubber, EPDM, silane cross-linked polyethylene, PE-Xb, electron-ray cross-linked polyethylene, PE-Xc, and copper) in flowing tap water showed that these pathogens develop in the biofilms and resist up to several weeks on EPDM and PE. *Legionella pneumophila* adheres to copper surfaces while *Pseudomonas aeruginosa* does not [181]. 

Among several polymeric pipes (PVC-C chlorinated polyvinyl chloride, PE-Xb, PE-Xc, PE-100, and PVC-P) tested for the transport of unchlorinated water, PVC-C is recommended for the control of biofilm growth. The ATP (adenosine triphosphate) concentrations, a measure of microbial activity in the biofilm, were minimum for PVC-C and maximum for PVC-P [199].

Among several tested pipes (stainless steel, glass, zinc-galvanized steel, copper, and polyvinyl chloride), *Mycobacterium avium* shows the highest adherence to galvanized steel and the smallest adherence to glass [227]. 

The total number of bacteria was determined by the Real-Time Quantitative Polymerase Chain Reaction (qPCR) method [195,196,228]. The detached biofilm was prepared as explained by Ren et al. (2015), and the number of pathogens was determined by cultivation on different media specific to each pathogen (plate-counting method). *Legionella pneumophila* requires more complex methods because other bacteria existent in the biofilm compete in the cultivation step. Therefore, an isolation step using heat and acid treatment is necessary prior to cultivation [229,230,231].

The efficiency of the disinfection process depends on the pipe material. Comparing chlorine disinfection in polyethylene and copper pipes, for the same disinfectant concentration, the microorganism presence decreases both in water and film on polyethylene pipe. For copper pipes, the effect of disinfection is weaker, being effective in the front part of the pipeline. This suggests that copper pipes require a higher disinfectant concentration for an efficient decrease of microbial numbers [232]. 

Antibiotics present in the water flowing in the DWDS contribute to the development of biofilm resistance to antibiotics [233,234]. It was found that at low concentrations, the antibiotics (tetracycline, sulfadiazine, and chloramphenicol) will enhance the growth rate of bacteria in the biofilms [192]. Antimicrobial resistance is given by microbes and pathogens that have genes resistant to the drugs developed to destroy them and can spread and multiply in the environment (water and biofilm). Although not enough long-term data is available, it is considered that this poses a potential health risk in humans [235]. Moreover, antibiotic resistance is often accompanied by metal resistance, meaning that the same bacteria that develop resistance to antibiotics also develop resistance to the metal presence [236].

## 4. Microplastic (MP) Fate in DWDS

In the recent years, the presence and fate of microplastics in drinking water has received increasing attention [237,238,239]. There are numerous MPs sources from various environments [240,241,242,243,244]. Once in DWDS, the MPs can sediment and be incorporated into the scale formed on the pipe inner surface. 

Variation in total MPs content (polypropylene, polyethylene, polystyrene, polyethylene terephthalate, and polyvinyl chloride) along a pipeline showed a decrease in MPs concentration in water from 1570.8 n⋅L^−1^ at water intake to 377.0 n⋅L^−1^ at the end of the pipeline network [245].

Attempts to remove MPs in conventional water treatment plants evidenced that this is not enough to reduce MPs. Although some retention of bigger particles takes place, there is also a fragmentation process, which generates even more small particles [244]. Negrete Velasco et al. (2023) evidenced the persistence in drinking water of some types of plastics (such as polyethylene and polyethylene terephthalate) after the water passage through Geneve’s water treatment plant [246]. Weber et al. (2021) found that the tap water from a German city did not contain MP particles bigger than 10 μm [247].

The toxicity of MPs derives from their chemical structure, from their physical and biological characteristics (small dimensions of different shapes that can adsorb chemicals can be sedimented or easily transported) [248]. 

**Figure 10:**
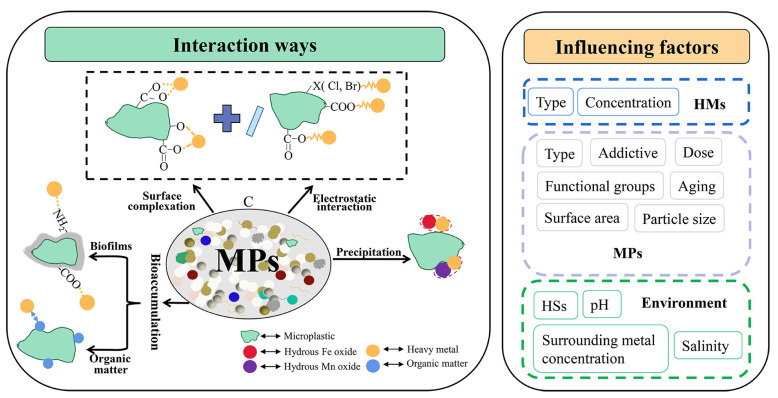
Interaction mechanisms between MPs and heavy metals present in the DWDS. Factors that influence the retention mechanisms (HMs—heavy metal complex) [249].

Furthermore, it has been reported that MPs (e.g., polyethylene, polystyrene, polyvinylchloride, polypropylene, polyamide, or polyformaldehyde) can interact with heavy metals (Cr, Co, Ni, Cu, Zn, Cd, Pb, Ag, Mn, and Hg) present in drinking water and contribute to metal accumulation [249,250,251,252] (Figure 10). 

In aquatic environments, such as DWDS, the interaction mechanisms between microplastics and heavy metals include:

- Electrostatic interactions and electrostatic interactions with surface complexation: Polar regions formed on corroded pipe surface interact with polar MPs surface. The polar surface of microplastics is given by different functional groups generated during MPs aging (e.g., the number of oxygen-containing groups increases with the MPs aging [253]). He et al. (2022) found that the pH and temperature influence the interaction between manganese ions and aged polystyrene microplastics. They proposed a mechanism based on the establishment of hydrated functional zone (an inner film, an outer film, and their enclosed space) [250]. The water chlorination underwent significant morphology O-functional group changes and created C-Cl bonds in MPs (polyethylene and thermoplastic polyurethane) [251]. This enhanced the aggregation ability of the MPs and their interaction with Cr(VI), which is a highly toxic compound.
- Sorption or bioaccumulation is enhanced by the biofilm developed on pipe or MP surface [254,255,256]. The interaction between metals and MP could be intensified in the warmer season because biofilm growth is much faster. This aspect requires more study to better understand the pipe–biofilm–MPs interactions.
- Electrostatic retention and bioaccumulation appear to be the main mechanisms of interaction between heavy metals and MP in aquatic environments [257,258,259,260,261,262]. More studies performed in set-ups that approximate the conditions from DWDS are needed to investigate how factors, such as pH, MPs size, water composition, and disinfectant type, influence the heavy metal–biofilm–MPs interaction.

It was found that the capacity of metal adsorption is different among polymers [257]. The adsorption capacity for cadmium ions decreases in the order PVC > PS > PP > PE.

MPs act as organic compounds carriers or adsorbents; they can accumulate dangerous chemicals present in small quantities in drinking water, such as polycyclic aromatic hydrocarbons (PAHs), polychlorinated biphenyls (PCBs), and organochlorine pesticides [252,263].

The accumulated MPs (containing heavy metals or dangerous organic compounds in larger quantities than in drinking water) can be later detach from the scale and be further transported by the flowing water, posing a health risk.

MPs present in water bodies are generally polyamide (PA, 33%), polyethylene terephthalate (PET, 15%), rubbers (10%), polyethylene (PE, 10%), and chlorinated polyethylene (CPE, 7%) [264]. Analysis of tap water showed MP particles present in almost half of the samples analyzed, and the polymers identified were polypropylene, polyester, and polyamide [265].

MPs were detected in different layers of the pipe scale: on the surface, in hard shell-like and porous core layers (Figure 5) [142,266]. The study was conducted using pipe scale samples collected from iron metallic pipes in use for about 20 years and polystyrene MP particles. The retention mechanism of MPs is complicated. For polystyrene, it was found that van der Waals, electrostatic interactions, hydrogen bonding, and pore filling were the main adsorption processes [266].

The scale accumulates MP particles (nylon and polyvinyl chloride) with sizes 50–100 μm while those higher than 200 μm flow with water [267]. 

The interaction between MPs and biofilm has not been extensively evaluated so far due to the complexity of the processes involved. The study of Chen et al. (2023) focused on the impact of hydraulic conditions on the development of MP-containing biofilm [193]. The MPs tend to form clusters in the biofilm with dimensions depending on the water flow rate (the higher the water flow rate, the lower the dimension of the cluster). At lower velocities (0.55 to 0.95 m/s), clusters bigger than 5 μm accumulate. Increasing the water flow velocity (1.14 m/s to 1.40 m/s) results in the accumulation of clusters with smaller sizes. Passing from stagnant to flow conditions can detach deposits from the wall pipe into the flowing water.

## 5. Mathematical Modeling 

The pipes affected by the leaching process were found to be metallic pipes and cement and cement-protected pipes. Leaching depends on the material pipe and the water characteristics (disinfectant dose, pH, if it is soft water). The compounds leached from the pipe surface will accumulate in water over the distance of the pipeline. Therefore, the concentrations of different dangerous compounds will be higher at the ends of drinking water supply pipeline sections. Few studies in the literature address the modeling and prediction of compounds leaching from different pipe surfaces. This is due to the complexity related to the processes and the fact that, depending on the type of pipe, the leaching processes are time-dependent. Hence, there is need for large spatial-temporal data to develop a model that predicts the leaching of pipeline components. 

Moreover, when developing a tool for modeling contaminant release from the pipe distribution system, it is necessary to include a model for microbial contamination and disinfection by-product (DBP) formation because these processes interfere with the release of contaminants from the pipeline [268]. 

Chlorine-based disinfectants react with natural organic matter and form DBP. DBP include THMs (trihalomethanes), HAAs (haloacetic acids), HANs (haloacetonitriles), HNMs (halonitromethanes), HAMs (halogenated acetamides), etc. Aldehydes (ALs) and iodinated DBP can be formed when water is disinfected by ozone and iodine [269].

The water distribution system is a highly dynamic environment, with changes occurring throughout the whole distribution system. Great attention is given to disinfection by-products in the water treatment plant, but few studies are dedicated to what happens afterwards and how DBP transform in the water distribution system. 

Because PVC-manufactured pipes are more susceptible to biological film formation, the mathematical model developed for these distribution systems must include the interaction between the organic matter and the disinfection substances.

Models to account for the longitudinal dispersion of pollutants in water distribution systems have been developed [270]. 

For a pipe system coated with cement, mathematical modeling of heavy metal (chromium and lead) release shortly after pipeline operation was studied by accounting for sorption [32,271]. By using this model, the chromium and lead concentrations at pipeline sections can be calculated after rehabilitation by cement lining. The developed mathematical model considers several mechanisms to describe the leaching of chromium and lead: dissolution, diffusion, advective transport, and sorption. The sorption included in the model considers the adsorption of heavy metals inside porous cement coatings. The diffusion accounts for chromium and lead transport in water phase in coating capillaries and in water phase inside the pipe. Experiments were performed in static [271] and in dynamic conditions [32]. Significantly different lead and chromium concentrations near the wall coating compared to the pipeline center were obtained by including diffusive transport in the liquid phase in the mathematical model [271]. The maximum cross-sectional average concentration depends on the pipe diameter, a pipe diameter twice as large will result in decreasing the maximum cross-sectional average concentration by half. 

The experiments performed under dynamic conditions used fresh water or water replaced periodically [32]. The water was characterized by its pH, alkalinity, and hardness. The calculated and experimental concentrations of lead and chromium were in good agreement. The calculated concentrations corresponding to a pipeline 30 km long reached values that are well below the threshold of the current regulations (2.5 mg/m^3^ for chromium vs. 25 mg/m^3^ in regulations and 2 mg/m^3^ for lead vs. 5 mg/m^3^ in regulations) [1]. However, these concentrations may occasionally be exceeded in case of water stagnation or if the flowing water is already rich in chromium or lead. The impact of biofilm and DBP formation was neglected in this model.

To prevent unplanned pipe failure, the lifetime of a piping system must be approximated. Because pipes are affected by flowing water, soil, and environmental characteristics, it is difficult to predict what will be the degradation along a pipeline. In this case, estimation of probability of failure based on Monte Carlo simulations may help. This method was applied in the case of AC pipe distribution system [272]. Considerable experimental data are needed to calculate the degradation rate for different sections of an AC pipe system (different pipe diameter sizes, water with different characteristics from different locations in Thailand). Monte Carlo simulations will provide several failure cases that will allow scheduling a pipe replacement program.

**Figure 11:**
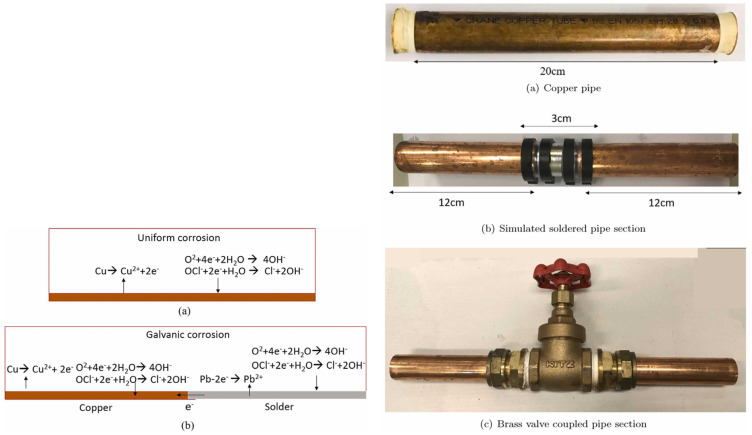
Schematic diagram of corrosion reactions of **left**: (**a**) uniform corrosion of copper pipes and (**b**) galvanic corrosion system with the half-cell reactions; **right**: (**a**) copper pipe section, (**b**) soldered copper pipe section and (**c**) brass valve coupled with copper pipe [45].

Using 3D computed fluid dynamics calculations, the influence of pipeline geometry, sampling methods, and the flowing regime on the lead concentration in drinking water was studied [44]. The case includes a system of copper pipes connected by lead solders and brass valves. It was found that galvanic corrosion takes place when lead is in contact with copper in the presence of water. The galvanic corrosion is an electrochemical process in which one metal corrodes when it is electrically connected with a dissimilar metal in the presence of a conductive liquid. In this case, lead is the anode and corrodes, while copper is the cathode (Figure 11). In case of stagnant water, the lead concentration increases and when the water is used again, concentration peaks of lead in tap water are generated [45]. The model is based on Ohm’s law, Faradays’ law, and polarization theory to predict lead concentration in stagnant water after a given time. The model does not include the effect of pH, temperature, and the concentration of organic and inorganic matters. To be used for modeling studies using water with different properties, the model must be adapted. The additional mass transport equations must be included to adapt the model to water flowing conditions. This model can be, however, adapted to explore the effect of other pipe materials, such as iron or stainless steel. 

The copper release in drinking water is controlled by the acidity of the flowing water rather than by the concentration of dissolved oxygen [107]. In stagnant water, copper corrosion and, therefore, copper release in drinking water is enhanced by the presence of hydrogen ions, which, in turn, increases the consumption of the dissolved oxygen. The kinetic modeling of dissolved oxygen consumption rate evidenced that for new pipes and low carbonate concentration, a zero-order linear kinetic rate law effectively describes the experimental data. For aged copper, the kinetics of dissolved oxygen consumption is better described by a first-order kinetic rate law. 

## 6. Conclusions

Metal-based pipes form scale, which contains and accumulates heavy metals (e.g., lead, vanadium, chromium, copper, arsenic, zinc, cadmium, and manganese) from the environment. The scales can detach and end up in the flowing water. Cement-based pipes release chemicals after commissioning. Calcium, aluminum, chromium, lead, and cadmium are the main ions released by cement-containing pipes. In the case of asbestos-containing cement pipes, asbestos fibers can be released into drinking water. Polymeric pipes are susceptible to releasing organoleptic substances (e.g., alkylphenols, aldehydes, ketones, and organotins generated or used in the manufacture process) within first weeks after the commissioning. 

The efficiency of the disinfection process depends on the pipe material. Metallic pipes (e.g., copper) may require a higher concentration of disinfectant. Regarding the corrosion process, there is a clear advantage of polymeric-based pipes versus metal-based pipes. The polymeric pipes do not develop scale deposits. They are, however, subjected to degradation by chain scission in the presence of water disinfectants that generate free radicals (e.g., ClO_2_).

All types of pipes develop biofilm on their surface. This biofilm retains and accumulates microplastics and compounds from water. Moreover, the biofilm accelerates the proliferation of opportunistic pathogens and bacteria. When changing the flow conditions or water parameters, the biofilm can detach, causing an instant increase in some compound concentrations that may pose a health risks (especially heavy metals and microplastics). Microorganisms present in the biofilm enhance metal-based pipe corrosion (microbiologically induced corrosion), making them even more vulnerable to mechanical failure and accelerating the release of dangerous compounds in drinking water. 

Changing from flowing to stagnant conditions will cause an enhanced corrosion of metal-based pipes. For the polymeric pipes, there are no studies that discuss this aspect, but it is expected that the stagnant conditions will affect the microorganism community in the biofilm.

Several approaches could help reduce the pollutants that otherwise will end up in drinking water:

- Eradication of lead from DWDS (pipes, lead solder, and high lead–copper alloy fittings), this requires a big financial effort and stronger regulatory directives.
- Because asbestos fibers are released from corroded AC pipes, it is necessary to speed up the replacement process of asbestos-containing pipes, with asbestos-free pipes.
- More frequent inspections and earlier replacement of iron-containing pipes when advanced corrosion is observed.
- Where corrosion is expected, corrosion inhibitors should be used (e.g., orthophosphate). However, their impact on biofilm development must be known.
- Adjust the disinfectant dose according to the type of pipes in the DWDS. Disinfection requires a compromise between the inactivation of pathogens and the formation of hazardous DBPs.
- Minimize the episodes of stagnant water, which are favorable to corrosion intensification, biofilm growth, accumulation in the scale of heavy metal ions and MPs from drinking water.
- The DWDS consist of several materials with different corrosion properties, thereby, different demands on water quality, disinfectant, and corrosion inhibitor. Overlapping different corrosion-controlling strategies for DWDS may result in unexpected results if the materials are very different.
- Implement a continuous monitoring system to maintain the water parameters in the limits fixated by water directives.

## Figures and Tables

**Table 1:** Human health effects of drinking water contaminants.

Contaminant	Health Effects Reported after Long-Term Exposure to Water Contaminated above Maximum Level
Aluminum	Risk factor for the development of Alzheimer’s disease [19]. Ranked number 188 in the ATSDR (Agency for Toxic Substances and Disease Registry) 2023 substance priority list [35].
Antimony	There is evidence for carcinogenicity by inhalation, no data available to indicate carcinogenicity by oral ingestion [36]. Ranked number 236 in the ATSDR 2023 substance priority list [35].
Asbestos	Link between the ingestion of asbestos fibers and gastrointestinal cancer [23]. Ranked number 96 in the ATSDR 2023 substance priority list [35].
Arsenic	Ingestion of arsenic through drinking water is related to the development of cancer at several sites in humans [30]. Ranked number 1 in the ATSDR 2023 substance priority list [35].
Cadmium	Kidney is the main organ where Cd accumulates, creating damage [34]. Ranked number 7 in the ATSDR 2023 substance priority list [35].
Copper	Gastrointestinal effects, possible metabolic disorders [37]. Ranked number 120 in the ATSDR 2023 substance priority list [35].
Chromium hexavalent	Carcinogenic [31] Ranked number 17 in the ATSDR 2023 substance priority list [35].
Lead	Neurodevelopmental effects, mortality (mainly due to cardiovascular diseases), hypertension, negative effects on renal function and fertility, and adverse pregnancy outcomes. Impaired neurodevelopment in children at lower dose exposure [11]. Ranked number 2 in the ATSDR 2023 substance priority list [35].
Manganese	Central nervous system is affected by long-term exposure to manganese; Gastrointestinal absorption of manganese is higher in children than adults [38]. Ranked number 143 in the ATSDR 2023 substance priority list [35].
Vanadium	Unknown effects to date, could be genotoxic, i.e., interacts directly with DNA [39]. Ranked number 208 in the ATSDR 2023 substance priority list [35].
Vinyl chloride	Linked to cancer occurrence in humans, genotoxic, i.e., interacts directly with DNA [40]. Ranked number 4 in the ATSDR 2023 substance priority list [35].

**Table 2:** Contaminants that can be leached in drinking water.

Pipe Type	Contaminant
Lead	hydrocerussite (Pb_3_(CO_3_)_2_(OH)_2_) [51,52,53,54,55,56] ions of V, Sb, Cu, Mn, and Cr [56,57,58,59,60]
Cement	Asbestos Ions of Ca, Al, Cr, Pb, and Cd [81,87]
Copper	Copper oxides [102,103,104,105,106]
Iron	Iron scale can accumulate other heavy metals from the flowing water (V, As, Cr, Mn) [132,137,138,139,140]
Steel	Ions of Cr, [80]; Mn > Fe > Zn > Pb [156]
Polymeric pipes	Release of organoleptic compounds [160] Can accumulate iron, manganese [166,167,168]

## Data Availability

Not applicable.
